# The emotional canvas of human screams: patterns and acoustic cues in the perceptual categorization of a basic call type

**DOI:** 10.7717/peerj.10990

**Published:** 2021-03-09

**Authors:** Jonathan W. M. Engelberg, Jay W. Schwartz, Harold Gouzoules

**Affiliations:** 1 Department of Psychology, Emory University, Atlanta, GA, USA; 2 Psychological Sciences Department, Western Oregon University, Monmouth, OR, USA

**Keywords:** Screams, Emotional expression, Nonlinguistic vocal communication

## Abstract

Screams occur across taxonomically widespread species, typically in antipredator situations, and are strikingly similar acoustically, but in nonhuman primates, they have taken on acoustically varied forms in association with more contextually complex functions related to agonistic recruitment. Humans scream in an even broader range of contexts, but the extent to which acoustic variation allows listeners to perceive different emotional meanings remains unknown. We investigated how listeners responded to 30 contextually diverse human screams on six different emotion prompts as well as how selected acoustic cues predicted these responses. We found that acoustic variation in screams was associated with the perception of different emotions from these calls. Emotion ratings generally fell along two dimensions: one contrasting perceived anger, frustration, and pain with surprise and happiness, roughly associated with call duration and roughness, and one related to perceived fear, associated with call fundamental frequency. Listeners were more likely to rate screams highly in emotion prompts matching the source context, suggesting that some screams conveyed information about emotional context, but it is noteworthy that the analysis of screams from happiness contexts (*n* = 11 screams) revealed that they more often yielded higher ratings of fear. We discuss the implications of these findings for the role and evolution of nonlinguistic vocalizations in human communication, including consideration of how the expanded diversity in calls such as human screams might represent a derived function of language.

## Introduction

Screams show remarkable evolutionary conservation across a broad range of species, including humans. Acoustically similar vocalizations occur in phylogenetically diverse taxa, usually when an animal is captured by a predator and faces imminent death (Högstedt, 1983). These screams might function to startle the predator, elicit mobbing behavior from conspecifics, warn kin, and/or attract other predators to increase the probability of the caller’s escape (Collias, 1960; Högstedt, 1983; Møller & Nielsen, 2010; Rohwer, Fretwell & Tuckfield, 1976; Toledo, Sazima & Haddad, 2011). It is likely that certain, widely conserved acoustic characteristics of screams were selected for in this context. Thus, screams across species share high amplitudes and wide frequency ranges, enhancing their propagation over distances and listeners’ abilities to locate them (Högstedt, 1983).

In several primate species, the contexts in which screams occur have diversified, shifting from limited predator-prey interactions to more complex and socially nuanced agonistic conflicts among conspecifics, where screams function to recruit aid from allies (Bergman et al., 2003; Bernstein & Ehardt, 1985; Cheney, 1977; de Waal & van Hooff, 1981). In the process, selection has also promoted acoustic diversification, such that different classes of screams tend to correlate with contextually relevant elements of the encounter, such as whether an attack comes from a dominant group member or from a lower-ranking animal involved in a rank challenge (macaques: Gouzoules, Gouzoules & Marler, 1984, 1986; Gouzoules, Gouzoules & Tomaszycki, 1998; Gouzoules & Gouzoules, 2000; vervets: Mercier et al., 2019; chimpanzees: Slocombe & Zuberbühler, 2005, 2007; Slocombe, Townsend & Zuberbühler, 2009), likely because clearer communication of these details was evolutionarily advantageous for both screamers and listeners. More recently, we have reported acoustic variation within some classes of rhesus monkey screams that is more directly attributable to differences in arousal level (or activation/alertness; Schwartz, Engelberg & Gouzoules, 2020), suggesting that variation in emotion is one proximate mechanism through which acoustic diversification has occurred. Intriguingly, screams have seen even greater contextual diversification in humans, where they are associated with a variety of emotional contexts including fear, anger, surprise, and even happiness (Anikin & Persson, 2017), although whether listeners perceive different emotions from screams remains unknown. This feature of human screams, suggesting novel functions in this evolutionarily conserved call type, render them a fascinating subject for understanding nonlinguistic emotional communication in our species.

The acoustic findings in nonhuman primate screams illustrate an important principle that has long guided research in animal communication, namely, that variation within call types, that is, graded differences in the spectrotemporal structure of a canonical call type such as a scream, often correlates with socioecologically relevant information and is perceptually salient to listeners (Gouzoules, Gouzoules & Marler, 1985, 1986; Fischer, 1998; Manser, Bell & Fletcher, 2001; Rendall et al., 1999). This variation is distinct from variation between call types, that is, the usage of one species-typical vocalization versus another, for example, screams versus other call types such as threats or predator alarm calls. Researchers usually conceptualize this variation as a constellation of vocal output in acoustic space, wherein exemplars varying within a call type cluster together (the centroid of which might represent a prototypical exemplar) and the boundaries between types are often fuzzy, not discrete (Fischer, Wadewitz & Hammerschmidt, 2017). For bioacousticians, determining these boundaries and disentangling the variation that separates call types versus the variation that exists within a type is a complex and ongoing task (Fischer, Wadewitz & Hammerschmidt, 2017; Schwartz, Engelberg & Gouzoules, 2019), one to which research within call types such as screams contributes.

Importantly, the factors determining acoustic variation within versus between call types are not likely to be identical, nor, by extension, are their functions or the information made available (Schwartz, Engelberg & Gouzoules, 2020). For example, in many nonhuman animals, variation within call types seems closely linked to the caller’s arousal level, whereas additional cognitive processes (e.g., strategic decision-making; Silk, Seyfarth & Cheney, 2016) appear to control the decision of which call to use, and whether or not to call at all. Some authors have consequently proposed that, of the two kinds of acoustic variation, the sort associated with within-call types is probably the closer correlate of a primate’s emotional state (Schamberg, Wittig & Crockford, 2018; Schwartz, Engelberg & Gouzoules, 2020).

This perspective, with its emphasis on call types and the significance of acoustic variation within each, has gained attention in the literature (Schwartz & Gouzoules, 2019; Wood, Martin & Niedenthal, 2017), but remains underexplored in our species. Historically, the majority of research on vocal emotional communication in humans has eschewed call types altogether, focusing instead on the capacity for prosodic cues accompanying speech to convey emotion (Juslin & Laukka, 2003; Scherer et al., 1991) and other types of information (Filippi, 2016; Tzeng et al., 2018). More recently, researchers have increasingly and promisingly investigated nonlinguistic vocalizations such as laughs, cries, and screams (Engelberg, Schwartz & Gouzoules, 2019; Hawk et al., 2009; Sauter et al., 2010; Schröder, 2003; Schwartz, Engelberg & Gouzoules, 2019)—that is, the vocalizations most likely comprising the repertoire of human call types (Anikin, Bååth & Persson, 2018) that are homologous to calls in other species (Davila-Ross, Owren & Zimmermann, 2009; Lingle et al., 2012; Owren, Amoss & Rendall, 2011). Much of this work has assessed the capacity for these vocalizations to convey emotion, revealing that different emotions map onto statistically-dissociable acoustic profiles (Sauter et al., 2010), and that listeners can distinguish rather nuanced categories of emotion (Hawk et al., 2009; Parsons et al., 2014; Sauter & Scott, 2007; Sauter et al., 2010; Schröder, 2003; Schwartz & Gouzoules, 2019; Simon-Thomas et al., 2009; Young et al., 2017). Indeed, some evidence suggests that nonlinguistic vocalizations may enable more accurate judgments about emotion than does prosodic speech (Hawk et al., 2009).

Still, in many of these studies, researchers have not defined or delineated the acoustic types represented among the stimuli, except for the constraint that they contained no identifiable words (Schröder, 2003). Stimuli have typically comprised a wide variety of different vocalizations (e.g., cries, laughs, screams and others in a single study, commonly under the umbrella term “affect bursts”; Scherer, 1994) from which listeners make emotional judgments. Thus, research has disproportionately explored variation between types—or else failed to distinguish call usage from within-type variation—and therefore has largely neglected a potentially distinct informational and functional channel. Where exceptions exist, the benefits of investigating this kind of variation are clear, most notably in the relatively robust literatures on human laughter (Bachorowski & Owren, 2001; Bryant & Aktipis, 2014; Todt & Kipper, 2001; Provine, 2000; Szameitat et al., 2009a, 2009b; Wood, Martin & Niedenthal, 2017) and infant cries, for which researchers have documented extensively the sources and perceptual effects of acoustic variation (Zeifman, 2001). For example, infant cries are not homogeneous distress signals, but instead vary acoustically according to a vocalizer’s level of distress (Bellieni et al., 2004; Koutseff et al., 2018; Porter, Miller & Marshall, 1986), influencing listeners’ perceptions of urgency and aversiveness (Cecchini, Lai & Langher, 2010; LaGasse, Neal & Lester, 2005; Protopapas & Eimas, 1997)—perceptions that in turn correlate with differential activation in cry-responsive areas of the brain (Li et al., 2018; Mascaro et al., 2014). Several other, biologically significant call types, including screams, remain relatively enigmatic. In particular, although an earlier study showed that longer or higher-pitched screams are perceived as more emotionally intense (Schwartz & Gouzoules, 2019), the perception of emotional information beyond intensity from contextually diverse screams remains unexamined.

In the present study, participants listened to a series of screams and rated their agreement with prompts corresponding to six different emotions: (1) anger or aggression (hereafter, anger); (2) fear; (3) frustration or annoyance (hereafter, frustration); (4) happiness or excitement (hereafter, happiness); (5) pain; and (6) surprise or startle (hereafter, surprise). These scales were selected on an exploratory basis to represent a variety of contexts in which human screams are naturally observed (Anikin & Persson, 2017). We favored a multidimensional ratings design over a forced-choice categorization because we predicted that different scream variants might express not only very disparate emotions, but possibly also variegated blends of emotion (Cowen et al., 2019). Our design enabled assessment of how listeners’ perceptions reflected this potentially nuanced emotional canvas communicated by human screams.

For this manuscript, we adopt the view that emotions are temporary multicomponent internal states that organize correlated responses in physiology, behavior, and (at least in humans) subjective experience (Paul & Mendl, 2018; Schwartz, 2020). A long-persisting debate (reviewed in Scarantino & Griffiths, 2011) concerns whether different emotions are subserved by biologically distinct systems (i.e., basic emotion theory; Ekman, 1992; Izard, 2007) or result from differential activation along a few underlying dimensions (i.e., dimensional theories; Barrett, 2006; Russell, 2003). The present study entailed ratings of agreement with emotion prompts mapping onto colloquial emotion categories, but we are agnostic as to whether the emotional states referred to by these prompts are discrete or biologically basic (as opposed to falling along dimensions).

Our primary aim was to determine whether screams yield consistent judgments about emotion across participants. This entailed, first, characterizing participants’ levels of agreement; if screams convey information about the emotions typically associated with different experiential contexts, listeners should achieve a non-trivial consensus. Second, we examined the correlational structure between ratings on the emotional prompts, enabling some assessment of how parallel or orthogonal were listeners’ judgments on each of the scales. Two non-correlated scales would suggest that listeners made a distinction between those two emotional contexts. On the other hand, two highly correlated scales might suggest they treated those contexts equivalently or judged them based on correlated cues.

An additional aim was to characterize the acoustic variation among screams that accounted for listeners’ ratings of emotion. Although many studies have examined the acoustic predictors of emotional judgments from speech (Scherer, Johnstone & Klasmeyer, 2003), the extent to which these findings apply to variation within nonlinguistic vocalizations is less clear. For example, Szameitat et al. (2009b) found that patterns of emotional expression in laughter were generally similar, but not equivalent to, those reported in speech. Aside from our earlier study examining the roles of scream pitch and duration in judgments of emotional intensity (Schwartz & Gouzoules, 2019), to our knowledge, there are no published investigations of emotion perception from acoustic variation existing within the human call type comprising screams.

We conducted a targeted acoustic analysis based on a set of six parameters that we hypothesized might influence the perception of emotion from screams. These included the mean and range of the fundamental frequency (F0; a measure of vocal fold oscillation rate that is typically perceived as pitch), as well as duration, mean HNR (a measure of periodicity, where lower values correspond to noisier sounds and higher values correspond to “purer”, tonal percept), loudness, and roughness. Variation in F0, duration or similar temporal parameters, and HNR are important cues for distinguishing emotions in prosodic speech (reviewed in Juslin & Laukka, 2003; Scherer, Johnstone & Klasmeyer, 2003) and nonlinguistic vocalizations (Raine et al., 2018; Sauter et al., 2010; Szameitat et al., 2009b, 2011; Schwartz & Gouzoules, 2019) and are also consistently linked to emotional variation in nonhuman mammals (Briefer, 2012; Briefer et al., 2015, 2019; Friel et al., 2019; Morton, 1977). The perceived loudness of a vocalization also potentially correlates with the perception of emotional variation (Scherer, 2003), but some of the variation in the loudness of our stimuli originated from factors other than the vocalizations themselves (e.g., different recording conditions). Thus, we included this measure to account for possible effects of loudness unrelated to the acoustic variation of interest. Finally, roughness corresponds acoustically to rapid amplitude modulation and perceptually to a harsh, “buzzing” quality in sounds (Arnal et al., 2015; Vassilakis, 2007). Arnal et al. (*ibid*.) proposed that roughness is a defining characteristic feature of screams that is absent from regular speech (but perhaps not from all other nonlinguistic vocalizations; Schwartz, Engelberg & Gouzoules, 2019; Li et al., 2018), and showed that rough screams are perceived as more fearful than screams artificially filtered to remove rough modulation. However, it is unknown how natural variation in roughness among screams, such as that documented by Schwartz, Engelberg & Gouzoules (2019), affects emotional perception.

A final aim of this study was to investigate whether ratings on emotion prompts varied as a function of the scream’s original source context. Specifically, we hypothesized that if screams have the potential to convey information about emotional context, participants should tend to rate them more highly on trials where the emotion prompt matched the source context. Due to a small number of stimuli in some contexts (Table 1), we could not formally investigate the effects of all specific source contexts on ratings, with the exception of happiness, a case of special interest given its potential uniqueness to human screams.

**Table 1:** Scream descriptives by original emotional context.

Original source context	*n*	Duration (s)	Mean F0 (kHz)	*F*0 Range (kHz)	Mean HNR	Mean roughness	Loudness (LUFS)
Anger	2	2.112	0.353	0.085	2.240	39.135	−22.505
Fear	5	1.319	1.426	0.963	5.908	34.374	−20.408
Frustration	1	0.296	1.940	1.062	7.610	30.068	−13.980
Happiness	11	1.522	2.165	1.244	12.909	26.702	−9.780
Pain	6	1.301	0.677	0.588	7.910	27.776	−18.662
Surprise	5	0.298	1.000	0.609	6.784	27.889	−19.448

## Materials and Methods

Testing took place over a two-year period at Emory University’s Bioacoustics Laboratory in the Department of Psychology. This research was conducted in compliance with Emory’s Institutional Review Board under IRB00051516, approved July 26, 2011.

### Participants

A total of 182 participants from Emory University took part in this study (124 female, 58 male; Age *M* = 19.418, SD** = 2.041). We aimed for a relatively large sample size because, for the questions explored here, the literature was sparce and thus precedents were few. Variation within screams remains largely undocumented and we therefore wanted to uncover even small effects (while appropriately noting when effect sizes are small). Participants were recruited via an online portal system and received class credit for completing the study. All participants provided their voluntary and informed written consent.

### Stimuli

Stimuli were selected from an in-lab corpus of screams collected from movies, television programs, advertisements, YouTube videos, and commercial sound banks (Human Sound Effects, Partners In Rhyme, Inc., Santa Monica, CA, USA; The Nightingale Voice Box, Nightingale Music Productions, North York, ON, Canada).

Screams were selected on the basis of sound quality (i.e., minimal noise; no overlapping sounds), and to represent a variety of emotional contexts. Original source contexts were identified (by HG) based on the surrounding socioecological circumstances and situational cues. These contexts are conventionally associated with a particular emotional state (hence the term *emotional context*), and it is likely that most vocalizers on average experienced the associated emotion, but we do not assert, nor for the purposes of this study is it necessary, that every instance of that context ineluctably entails that emotion (for example, in acted renditions). We chose instead simply to identify the context because of the greater certainty and objectivity in making these distinctions. Indeed, that professional actors’ screams are sometimes indistinguishable from naturally occurring ones (Engelberg & Gouzoules, 2019) might suggest that an authentically experienced emotional state is not a prerequisite to produce a credible scream. That said, listeners themselves were asked to make judgments about the likely associated emotion, and not the surrounding production context. Thus, our references to context in this article concern only the source categorizations and not the perceptual judgments made by participants.

The six contexts to which screams were categorized were the basis for the six prompts listeners were presented in the study. These contexts were deemed appropriate for *best* capturing the emotional situations in which each scream occurred. We reasoned and predicted that participants’ responses would reflect the emotions expressed by the screams and thus the situational contexts in which they were produced, but we recognize that some of the screams might involve contexts associated with multiple emotions, a hypothetical example being the fear and pleasurable excitement associated with a rollercoaster ride. Although we considered including screams from additional contexts (e.g., embarrassment or sex), we limited this study to six so as not to induce fatigue or task disengagement in participants, who were asked to rate all screams on all six scales for a fully-crossed experimental design.

Stimuli were processed as previously described in Engelberg, Schwartz & Gouzoules (2019). Specifically, online videos were captured or downloaded using Total Recorder version 8.0 (High Criteria, Inc., Richmond Hill, ON, Canada) and WinXHD Video Converter Deluxe (Digiarty Software, Inc., Chengdu, China), while DVD media were extracted using WinXDVD Ripper Platinum (Digiarty Software, Inc., Chengdu, China). All source videos were saved, converted to the MPEG file format, and cropped at timestamps surrounding the target vocalizations.

Audio files were extracted and converted to 16-bit 22.05 kHz WAV files using Adobe Audition CC (Adobe Systems, San Jose, CA, USA) and Audacity version 2.1.2 (http://audacity.sourceforge.net). Edits were applied when necessary to delete any clicks and pops (Owren & Bachorowski, 2007) or mitigate noise without distorting or interfering with the acoustics of the screams themselves (as determined by listening and by visual inspection of spectrograms). Additionally, in the case of some DVD sources, separate tracks containing background music were removed. Any screams that would have required more extensive editing were not used in the stimulus set.

The final stimulus set consisted of 30 screams from 26 different vocalizers. Again, the total number of stimuli was intentionally limited to reasonably obtain ratings on each scale from every participant and for every scream, enabling a fully-crossed design (i.e., one observation per prompt for each combination of participant and stimulus) and a large number of raters per stimulus. Female vocalizers produced 22 of the screams while males produced eight. The imbalance in gender representation was the consequence of our goal to include an ample number of screams from happy emotional contexts, which proved difficult to find for males (an observation we suggest is noteworthy). No differences were found in ratings on any prompt between male and female participants across all screams, nor between their ratings across only female screams or only male screams (Independent sample *t-*tests, *p* > 0.07 in every case, with the exception of ratings of frustration on male screams, which were not significant when corrected for the number of tests). The distribution of original source contexts is presented in Table 1 along with means for each measured acoustic parameter (see “Acoustic Analysis” below).

### Procedure

The experiment was conducted on E-Prime 2.0 software (Psychology Software Tools, Inc., Pittsburgh, PA, USA) running on a Sony VAIO Pentium 4 computer (model PCV-RS311). Participants were instructed to listen to screams and indicate their level of agreement with statements appearing on-screen.

The start of a trial was indicated by the word “Ready” appearing in the center of the screen. After a period of 0.50 s, a stimulus was delivered through headphones (JVC G-Series model HA-G55, JVCKENWOOD USA Corporation, Long Beach, CA) while the screen displayed a prompt related to the emotional state of the vocalizer (e.g., *“Rate your agreement with this sentence. This person is FRIGHTENED.”)*. Participants used the computer mouse to indicate their agreement with the prompt by clicking on-screen buttons labelled with the numbers 1 through 5, with 1 indicating strong disagreement and 5 indicating strong agreement. (Listeners may have interpreted 3 as neutral, undecided, or uncertain, indicating neither disagreement nor agreement, although it was not labelled as such.) After participants selected a response, the screen displayed their choice (e.g., “You chose 3.”) and an interval of 1 s proceeded before the next trial.

Each scream was presented 6 times throughout the experiment, resulting in 180 total trials (six presentations × 30 screams), with each presentation requiring the participant to judge the scream on a different emotion prompt: (1) aggressive or angry; (2) annoyed or frustrated; (3) excited or happy; (4) frightened; (5) in great pain; and (6) surprised or startled. As noted above, these prompts were chosen to reflect the emotional contexts that were represented among the stimuli. We distinguished anger versus frustration, and therefore tested these in separate prompts, based on whether the eliciting event was other-caused (anger), and thus more associated with a possibility of physical threat, or circumstance-caused (frustration; Roseman, 1996). Similarly, we distinguished fear from surprise because fear is necessarily negatively-valenced, but not necessarily short in duration, whereas the reverse is true for surprise (Kreibig, 2010), distinctions that could conceivably result in differences in vocal production and perception. All participants judged every scream on all 6 of these prompts, except for very rare cases in which a participant accidentally proceeded by clicking on a part of the screen that did not correspond to a number (*n* = 148 out of 32,760 trials across all participants). Stimuli and questions were presented in a fully randomized order, with the exception that no individual scream was ever presented consecutively.

Participant information was collected from a short questionnaire following the experiment. This included information about gender and age, as well as information about native language, handedness, and experience with screams in media that was used in concurrently administered studies (Engelberg, Schwartz & Gouzoules, 2019).

### Analysis

#### Acoustic analysis

Six acoustic parameters were measured for each scream: duration, mean F0, F0 range, mean HNR, mean roughness, and Loudness Unit Full Scale (LUFS). Analyses were conducted on spectrograms generated from the waveforms by Fast-Fourier Transform (FFT) on Praat (Boersma & Weenink, 2013). Duration was measured with Praat’s selection tool by highlighting the spectrogram from vocalization onset to end, excluding any clear reverberation. Mean F0, F0 range, and mean HNR were measured using the Quantify Source command in the GSU Praat Tools script package (Version 1.9, Owren, 2008). This script estimates the sound’s F0 contour from a selected spectrogram segment using Praat’s To Pitch autocorrelation function, and estimates HNR using Praat’s To Harmonicity autocorrelation function. Measurements were made using a 50 ms analysis window, 75-Hz pitch floor, and 3500-Hz ceiling. The automatically generated F0 contour was checked manually for errors (e.g., spurious voiced segments or octave jumps) by visual and auditory comparison to the original spectrogram before obtaining measurements of mean F0, mean HNR, F0 minimum, and F0 maximum; F0 range was calculated as the difference between the latter two. Roughness was measured using the modulationSpectrumFolder function in the *soundgen* package in R (Anikin, 2019). Finally, loudness was measured as LUFS using the Amplitude Statistics function in Adobe Audition (Build 13.0.8.43; ITU-R BS.1770-4, 2015 loudness algorithms). LUFS is measurement of loudness employing algorithms adopted by the International Telecommunication Union (ITU-R BS.1770). The scale controls for the perception of loudness and includes weighting to account for differences in frequency response (humans are much more sensitive to volume changes in mid-range frequencies compared to the high frequencies found in screams). The loudness unit is equivalent to a decibel, except that it is weighted to the human perception of audio rather than just measuring the electrical signal.

Descriptive statistics for these parameters are presented in Table 1. We note that representation of each source context in our stimulus set was not equal, nor the representation of potentially salient vocalizer characteristics (e.g., male vs. female; child vs. adult) within each source context. Thus, the acoustic means in Table 1 are meant only to serve as descriptors for the present stimulus set and not to draw conclusions about any true acoustic differences between the contexts.

#### Statistical analysis

Statistical analyses were performed using SPSS Statistics Version 25 (IBM Corp., Armonk, NY, USA) and the R statistics environment (R Core Team, 2018).

Levels of participant agreement were estimated using the intraclass correlation statistic (ICC, 2-way random model, single-score consistency). The ICC is an interrater reliability index applicable to datasets of more than two raters. The ICC model used here takes into account systematic biases between raters in use of the scales (Koo & Li, 2016). It is calculated based on the ratio of the variance of interest (here, the mean square between subjects minus the mean square of errors, divided by the number of raters) over the total variance in a data matrix (Liljequist, Elfving & Roaldsen, 2019). A separate ICC was calculated for each emotion prompt, that is, based on participants’ ratings of all 30 items in that prompt. Participants who did not judge all 30 items due to input error were not included in the calculation of that prompt’s ICC. Additionally, a separate ICC value was calculated from only the 30 items in which the prompt matched the original context (“match trials”). ICCs were compared by referencing their estimated 95% confidence intervals (Koo & Li, 2016).

Correlations between screams’ mean ratings on the emotion prompts were examined using a Pearson’s correlation matrix. To characterize this correlational structure further, a principal component analysis (PCA) was conducted on the screams’ mean ratings. The extracted components with Eigenvalues > 1 are described.

To analyze the effects of scream acoustics on ratings, six separate cumulative link mixed models (CLMMs) with a logit link were fitted using the clmm function in the *ordinal* package in R (Christensen, 2019), with ratings on each prompt as an ordinal outcome variable, the mean-centered acoustic parameters as fixed effects, and participant and stimulus as crossed random effects. Mean HNR and roughness were highly correlated (*r* = −0.703) and their inclusion in the same models resulted in high measures of collinearity (Variance Inflation Factors, VIFs > 6), which leads to inflated standard error estimates (Jaeger, 2008). Between the two variables, HNR yielded higher VIFs. Thus, to reduce collinearity, roughness was included and HNR was excluded from the models reported in this paper (see Lima, Castro & Scott, 2013, for a similar approach to correlated predictors). For confirmation, however, we repeated all analyses with HNR instead of roughness, which resulted in nearly identical significant effects as those reported for roughness (except in opposite directions, given their negative correlation). Maximum likelihood estimates and standard errors for each parameter were estimated using the Laplace approximation. The significance of each effect was determined using likelihood-ratio tests via the Anova function in the *car* and *RVAideMemoire* R packages (Fox & Weisberg, 2018; Hervé, 2020). Additionally, to determine the acoustic correlates of the principal components described above, two separate multiple linear regressions were conducted using the acoustic parameters as predictor variables and screams’ scores on each principal component as outcome variables. Again, due to high collinearity, mean HNR was excluded from these models.

To analyze whether a source context matching the rating prompt increased the likelihood of higher ratings, a CLMM was fitted with ratings as the outcome variable and match versus non-match coded as a binary predictor variable, with random effects for participant and stimulus. The significance of the match parameter was determined using a likelihood-ratio test. Finally, for the source context best represented among our stimuli, happiness (*n* = 11), we explored how participants’ ratings varied as a function of emotion prompt. To conduct this analysis, a CLMM was fitted using only trials with happiness screams (12,012 trials), with ratings as the outcome variable, emotion prompt as the predictor variable, and participant and stimulus as random effects. Post-hoc pairwise comparisons of least-square-means were then conducted to estimate individual effects of each prompt relative to each other, using the Kenward-Roger method with Tukey *p-*value adjustment via the *lsmeans* package (Lenth, 2016).

## Results

### Overall ratings and agreement

**Figure 1:**
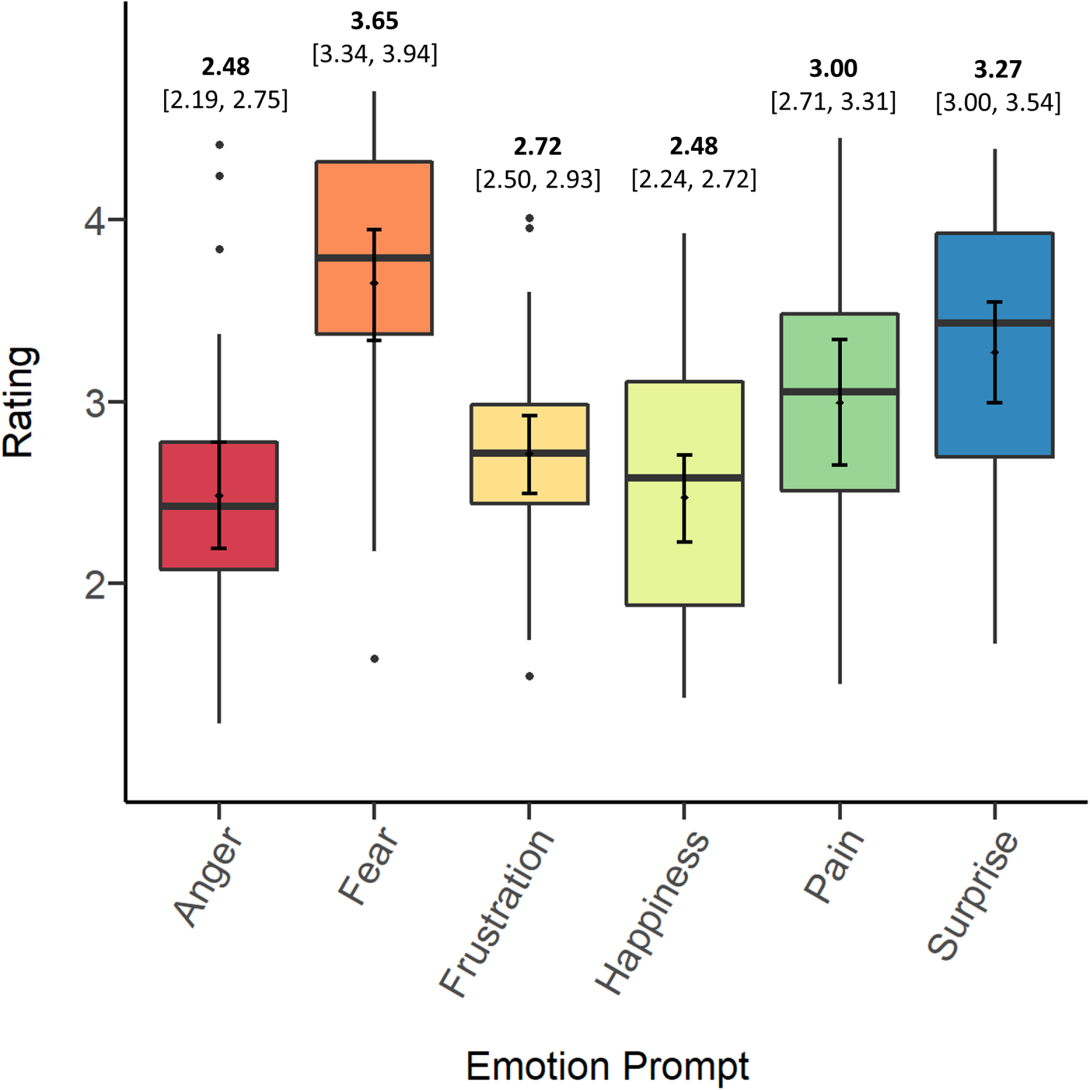
Means (bold) and confidence intervals (in brackets), medians and distributions of screams’ ratings on each of the emotion prompts.

Figure 1 depicts the means, bootstrapped confidence intervals, medians, and distributions of screams’ ratings on each prompt. In general, the data suggest that, independent of original context, participants tended to rate stimuli highest in fear, followed by surprise, pain, and frustration. Participants tended to rate screams lowest in anger and happiness. In the Discussion we consider several possible explanations for differences in overall ratings between prompts.

Table 2 presents participants’ intraclass correlations (ICCs) for each of the emotion prompts. All ICCs significantly exceeded chance (*F*-test, *p* < .001), indicating consensus among different raters. Per commonly used guidelines and descriptives for interpreting psychological data (Cicchetti, 1994; Hallgren, 2012), only ratings on fear and pain reached a “fair” level of agreement across all screams. However, overlapping confidence intervals warrant caution in the interpretation that participants agreed significantly more on any of the prompts.

**Table 2:** Participant agreement statistics.

Emotion prompt	ICC	95% CI
Anger	0.396	[0.292–0.544]
Fear	0.453	[0.343–0.601]
Frustration	0.241	[0.166–0.367]
Happiness	0.322	[0.230–0.464]
Pain	0.451	[0.341–0.598]
Surprise	0.373	[0.272–0.520]
Match	0.441	[0.332–0.589]

We suspected that participants might have reached a greater consensus on match trials, those in which the emotion prompt matched the original source context. To investigate this possibility, we calculated the ICC on match trials only and compared this value to the ICCs for each emotion prompt, each of which were mostly calculated from trials not matching the original context (for example, 25/30 screams that participants rated on fear were not from a fear context). The match ICC was higher than the ICCs for anger, frustration, happiness, and surprise, but lower than the ICCs for fear and pain, and again, confidence intervals overlapped in every case. Thus, it appears that participants did not tend to agree more on prompts matching the original source contexts.

### Correlations between emotion ratings

Pearson’s correlations were conducted on the screams’ mean ratings on each of the emotion prompts, indicating significant correlations between the scales (Table 3). Ratings of anger, frustration, and pain were highly positively correlated with one another and negatively correlated with ratings of happiness and surprise, which were positively correlated with each other. Ratings of fear did not correlate with ratings on any other emotion prompt.

**Table 3:** Pearson’s correlation matrix between mean ratings for the emotion prompts.

Emotion prompt	Anger	Fear	Frustration	Happiness	Pain	Surprise
Anger	1					
Fear	0.117	1				
Frustration	0.962*	0.136	1			
Happiness	−0.696*	−0.061	−0.675*	1		
Pain	0.768*	0.161	0.735*	−0.804*	1	
Surprise	−0.639*	0.255	−0.597*	0.709*	−0.860*	1

**Note:**

* Significant at *p* < 0.01.

To further characterize this correlational structure, we conducted a principal component analysis (PCA) on the correlation matrix, yielding two principal components with Eigenvalues greater than 1 that cumulatively accounted for 85.6% of the variance (Table 4). The first PC, accounting for 66.4% of the variance, loaded positively with ratings of anger, frustration, and pain, and negatively with ratings of happiness and surprise. The second PC, accounting for 19.1% of the variance, loaded positively with fear. Overall, this correlational structure suggests at least two dimensions of variation in participants’ ratings of emotions from screams, one separating the perception of (non-fear) negatively-valenced states (anger, frustration, pain) from positively or potentially neutrally-valenced states (happiness, surprise), and the other primarily separating the perception of fear from non-fear. Again, in the Discussion, we consider multiple explanations that might account for these general perceptual correlations.

**Table 4:** Results of PCA on mean ratings for the emotion prompts.

	Principal Component	
Variance Explained	1	2
Eigenvalue	3.986	1.147
% Variance	66.427	19.124
% Cumulative Variance	66.427	85.551
Component Matrix		
Anger	**0.915**	0.127
Fear	0.073	**0.971**
Frustration	**0.894**	0.158
Happiness	**−0.869**	0.023
Pain	**0.936**	0.035
Surprise	**−0.844**	0.403

**Note:**

PCs > 0.8 are bolded.

### Acoustic predictors of emotion ratings

**Figure 2:**
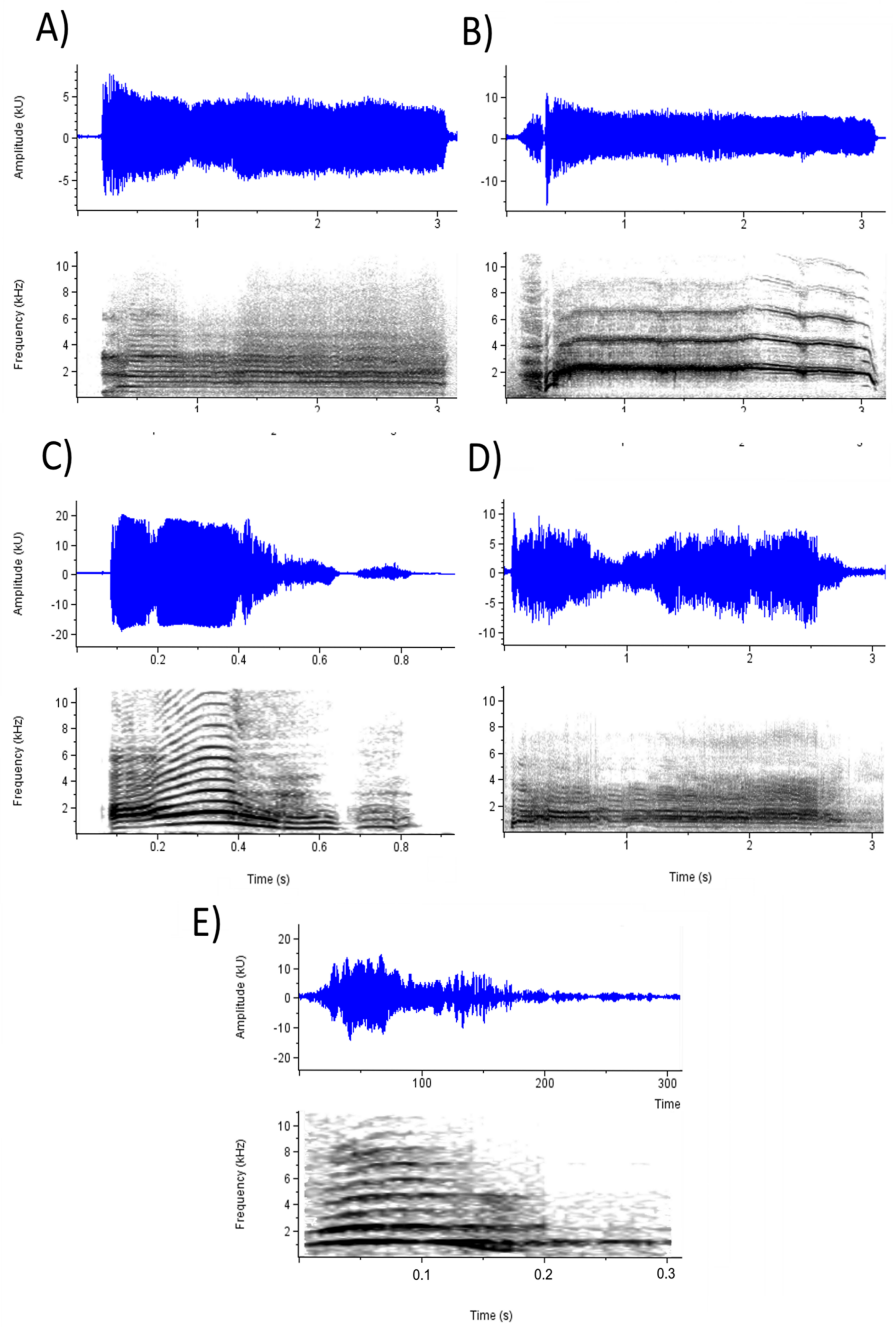
Waveforms and spectrograms depicting the screams with the highest ratings on each emotion prompt. (A) Anger (Mean rating = 4.41; Anger_2 in Supplemental Data) and Frustration (Mean rating = 4.01); (B) Fear (Mean rating = 4.70; Fear_5); (C) Happiness (Mean rating = 3.92; Pain_6); (D) Pain (Mean rating = 4.45; Pain_4); and (E) Surprise (Mean rating = 4.39; Surprise_2).

To investigate the effects of selected acoustic parameters on emotion perception, CLMMs were fitted using the parameters as fixed effects, subject and stimulus as crossed random effects, and ratings on each emotion prompt as outcome variables in six separate models. Table 5 presents the results for the acoustic models. Every parameter except F0 range and loudness significantly affected ratings on at least one emotion prompt and, when taking into account the direction of effects, most emotion percepts were influenced by a unique combination of parameters. Examination of these effects is facilitated with reference to Fig. 2, which depicts waveforms and spectrograms of the five screams with the highest mean ratings on (a) Anger and frustration, (b) Fear, (c) Happiness, (d) Pain, and (e) Surprise. Perception of anger and frustration was associated with screams of higher roughness (Fig. 2A). Perception of fear was associated with higher mean F0 (Fig. 2B; note the high F0 relative to the other spectrograms). Perception of happiness was associated with shorter duration and lower roughness (Fig. 2C). Perception of pain, like anger and frustration, was associated with higher roughness as well as longer duration (Fig. 2D). Finally, perception of surprise was associated with screams of shorter duration (Fig. 2E; note the short timescale).

**Table 5:** Acoustic predictors of emotion ratings.

Emotion prompt	Duration (s)	Mean F0 (kHz)	F0 Range (kHz)	Roughness	LUFS
CLMM estimates					
Anger	0.329 [0.182]	−0.178 [0.372]	−0.012 [.462]	0.210*** [0.038]	0.046 [0.042]
Fear	0.265 [0.194]	1.037** [0.398]	0.698 [0.494]	0.037 [0.040]	−0.015 [0.045]
Frustration	0.155 [0.128]	−0.150 [0.261]	0.088 [0.324]	0.141*** [0.027]	0.028 [0.029]
Happiness	−0.638*** [0.137]	0.292 [0.281]	−0.010 [−0.348]	−0.107*** [0.029]	0.037 [0.032]
Pain	0.892*** [0.203]	−0.419 [0.416]	0.018 [0.516]	0.130** [0.042]	0.059 [0.047]
Surprise	−0.854*** [0.142]	0.531 [0.289]	0.142 [0.359]	−0.054 [0.029]	−0.019 [0.033]
Principal components					
PC1	0.443*** [0.118]	−0.254 [0.241]	−0.0001 [0.299]	0.104*** [0.024]	0.018 [0.027]
PC2	−0.073 [0.139]	0.597* [0.284]	0.445 [0.353]	0.040 [0.029]	0.003 [0.032]

**Notes:**

* Asterisks indicate significance at *p* < 0.05.

** Asterisks indicate significance at *p* < 0.01.

*** Asterisks indicate significance at *p* < 0.001.

Brackets indicate 95% Confidence Intervals.

All significant effects are shown in bold.

The earlier referenced principal components from the perceptual ratings map substantially well to these acoustic results: the emotions that loaded onto PC1 generally shared distinguishing acoustic features, whereas fear, solely loading onto PC2, was also singular in its acoustic properties. To test this, we conducted two multiple linear regressions using the acoustic parameters as predictor variables and the principal component scores from PC1 and PC2 as outcome variables in two separate models (Table 5). These data showed that high PC1 scores, corresponding to higher ratings of anger, frustration, and pain and lower ratings of excitement and surprise, were predicted by longer duration and higher roughness. Higher PC2 scores, corresponding to higher ratings of fear independent of ratings on other emotion prompts, were predicted by higher mean F0s.

We note two important considerations when interpreting these data. First, although these acoustic results characterize the perception of emotions in our study, they might not necessarily correspond to the actual acoustic properties of screams expressing each emotion. For example, although only fear perception was associated with higher F0s, the screams with the highest F0s in our study were those produced in a happy emotional context (Mean = 2.17 kHz; see Table 1), which, perhaps, accounts for the finding that screams produced in contexts associated with happiness positively predicted the perception of fear (see section below).

**Figure 3:**
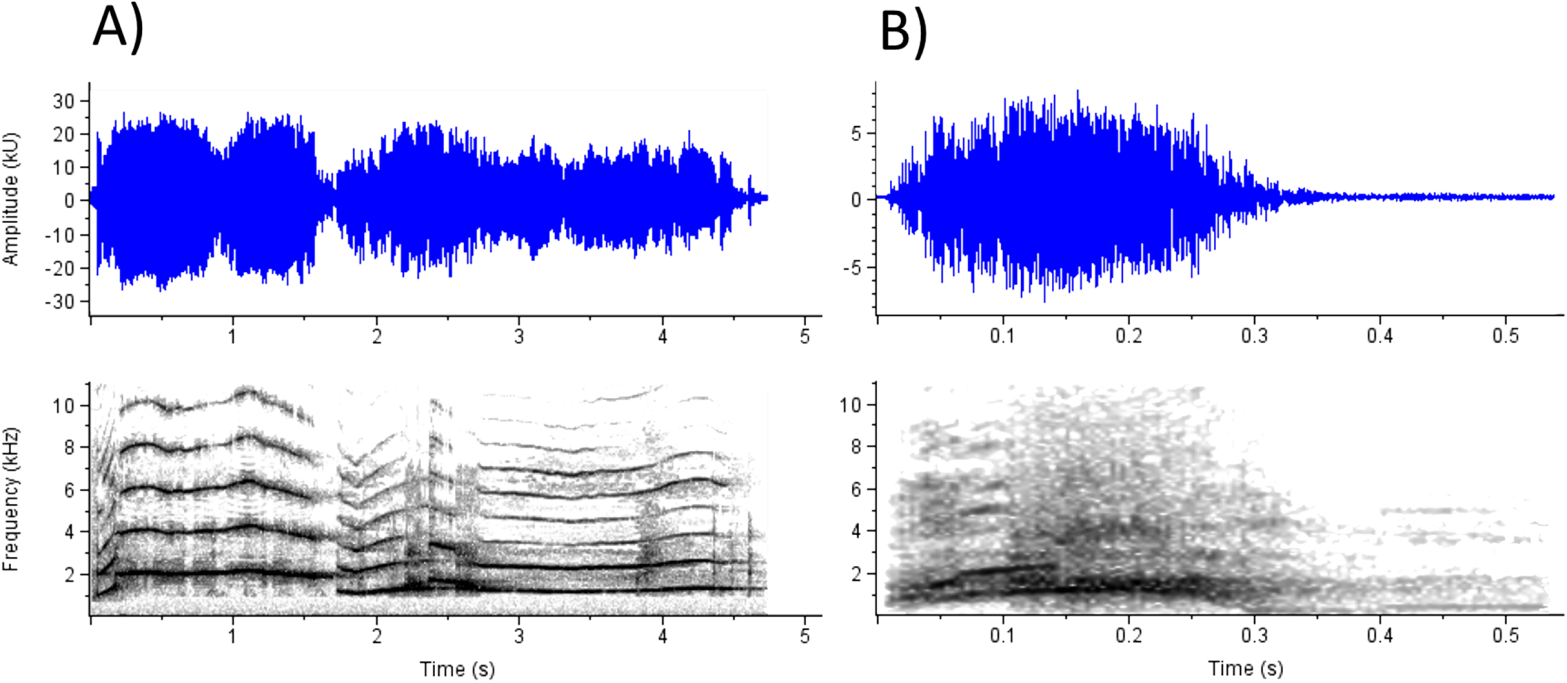
Two exemplars for which relationships between mean ratings across emotions do not adhere to the general correlations between scales. (A) A scream with low ratings on anger, happiness, and surprise, despite the generally negative correlation between anger with happiness and surprise (Happiness_3 in Supplemental Data). This scream also has rather different ratings for anger and pain, despite their strong positive correlation. (B) A scream with low ratings on happiness and high ratings on surprise, despite their strong positive correlation (Fear_3).

Second, although both the acoustic results and the correlation matrix between ratings on each emotion prompt suggest a broad, perceptual continuum with anger, frustration, and pain on one side, and happiness and surprise on the other, these relationships are not absolute or canonical, and the exceptions are worth examining (Fig. 3). Figure 3A depicts a scream with low ratings on anger despite simultaneously low ratings on happiness and surprise (with which anger ratings are negatively correlated) and high ratings on pain (with which anger ratings are positively correlated). This exemplar’s particularly long duration might explain its ratings of pain, happiness, and surprise. However, this scream is also low in roughness, and anger ratings were predicted by roughness to a greater extent than other emotions; thus the combination of long duration and low roughness might partially account for the exemplar’s idiosyncratic ratings. Similarly, Fig. 3B depicts a scream with somewhat lower ratings on happiness and higher ratings on surprise, despite the positive correlation between the two prompts. This scream is short (predicting higher ratings on happiness and surprise) but also rough and noisy (significantly predicting lower ratings on happiness only). Thus, although the perceptions of anger and pain, or happiness and surprise were correlated, listeners seemed to differentiate between these concepts and, in some cases, use acoustic information to differentiate their ratings between these scales. In other words, acoustic variation within screams likely allows for more heterogenous perceptions of emotion than the correlation matrix might suggest.

### Ratings as a function of source contexts

To investigate whether participants tended to provide higher ratings when the prompt matched the source context, we coded a binary match variable (match = 1 if the prompt matched the context, 0 if not) and used this as a predictor variable in a CLMM with ratings as the outcome variable, and stimulus and participant as random effects. This model revealed a significant positive effect of a matching emotion prompt (*β* = 0.869, SE = 0.0275, *p* < 0.001), indicating that participants generally provided higher ratings when a prompt matched the source context.

More close-grained analyses of ratings relative to most original source contexts were precluded because of the limited number of screams from each context. However, happiness screams were considered a point of special interest, given their potential uniqueness in humans, and they were the best represented source context in our stimulus set (*n* = 11). Therefore, in a qualified assessment of how participants interpreted happiness screams, we fitted a CLMM using the emotion prompt as a categorical fixed effect, subject and stimulus as crossed random effects, and happiness ratings as the outcome variable. This model revealed an overall effect of the prompt on ratings of happiness-associated screams (likelihood ratio test, *p* < 0.001). Post-hoc pairwise comparisons of least-square means with Tukey *p-*value adjustment were then used to examine the effects of each prompt relative to each other (Table 6). Although ratings on the happiness prompt were significantly higher than on the anger prompt, they were lower than ratings on fear, pain, and surprise prompts; fear prompts in particular tended to yield much higher ratings. These data suggest that happiness was not well recognized among our stimuli, and that fear perception included a subset of false positive assessments of screams associated with excitement.

**Table 6:** CLMM effects of prompts on ratings for screams from happiness-associated contexts.

	Anger	Fear	Frustration	Pain	Surprise
**Happiness**	0.45*	−2.352*	**0.042**	−0.551*	−0.897*
Anger		−2.802*	−0.408*	−1.001*	−1.347*
Fear			2.394*	1.801*	1.455*
Frustration				−0.593*	−0.939*
Pain					−0.346*

**Notes:**

* Asterisks indicate significance at *p* < 0.001.

Effects of happiness prompt relative to prompts associated with other emotions are shown in bold.

## Discussion

Screams are phylogenetically widespread, and although they are primarily associated with anti-predation across most taxa, the range of emotional contexts in which they occur has diversified over the course of primate evolution, especially for humans. With few exceptions (Schwartz & Gouzoules, 2019), potential relationships between listeners’ perceptions of emotion and the acoustic variation among screams have remained largely unexamined. In the present study we investigated listeners’ responses to screams associated with different emotional contexts using six emotion prompts. We found that listeners derived varied emotional meanings from the acoustic variation among screams. We also identified several acoustic parameters that significantly predicted perceptions on each emotion prompt. These results suggest that the contextual breadth of human scream usage is, to some extent, mirrored by an acoustic diversification that enables meaningful differentiation among scream variants.

### Agreement and perceptual dimensions

On every prompt, participants showed higher agreement than expected by chance. Depending on the prompt, levels of agreement ranged from “poor” to “fair” per the guidelines and terminology for interpreting reliability statistics (Cicchetti, 1994). We note, however, that these guidelines were developed for validating psychological assessment instruments (i.e., tests designed for reliability) and thus are likely to be especially stringent. Agreement on our prompts was comparable to those in other studies involving ratings of vocal emotional stimuli (Bänziger, Patel & Scherer, 2014). Given the constraints that participants here rated exemplars all belonging to the same basic call type, screams (Schwartz, Engelberg & Gouzoules, 2019), we suggest that their level of agreement is meaningful. It points to the significance of within-call type variation that, for screams, listeners reached non-trivial levels of agreement as to more specific emotional meanings.

On average, participants tended to rate stimuli highest in fear and lowest in anger and happiness. These differences in ratings might reflect that participants more commonly attributed fear to screams, and/or that they were more confident in their assessment of fear and thus tended to register higher ratings. Anger was one of the least represented emotions among screams’ original source contexts, so it is plausible that participants were accurate in less frequently perceiving anger among our stimuli. However, happiness was the most commonly represented original source context in our stimulus set, yet participants tended to perceive it less than other emotions. The section below, discussing ratings on each prompt for happiness-associated screams, explores these findings in greater detail.

Correlations between mean ratings on each prompt revealed that listeners perceived emotions from screams at least along two perceptual dimensions. First, their perceptions of anger, frustration, and pain generally grouped together while diverging from their perceptions of happiness or surprise, implying, at a minimum, basic differentiation between the perception of some clearly negatively-valenced states from non-negative states. Recall, however, that these dimensions describe variance in screams’ mean ratings, not their acoustic features, and that ratings of some exemplars suggest more fine-grained perceptual distinctions (Fig. 3). With the possible exception of anger and frustration, which likely have conceptual overlap, participants apparently differentiated between each emotion but judged some according to similar (but not perfectly correlated) cues, discussed below. The second dimension roughly mapped exclusively onto the perception of fear. Thus, some variance in the perception of fear appeared orthogonal to the perception of the other emotions, an interesting observation given the likely evolutionary significance of fear with respect to the origin of screams (although we note that even in fear contexts, the communication of fear itself might not have been the call’s primary adaptive function, which likely centered on startling, distracting, or driving away a predator).

An interpretation of these dimensions must take into account that our selection of original source contexts and prompts was preliminary and exploratory; our goal was to sample a variety of commonly recognized scream-associated contexts, and not discrete or archetypal representations of each emotional state, as the latter would require empirical evidence not yet available in the literature. Certain states such as anger and pain may often co-occur, further contributing to listeners’ correlated perceptions of these emotions. It is very likely that in natural contexts, vocalizers convey, and listeners perceive, blends of emotion (Cowen et al., 2019), a feature that would fit our notion of the emotional *canvas* of human screams.

### Acoustic predictors of emotion perception

We investigated the role of six acoustic parameters (mean F0, F0 range, duration, mean HNR, roughness, and loudness) in participants’ judgments on each emotion prompt. Specifically, we analyzed how these parameters affected listeners’ ratings without assuming these parameters are those that categorically delineate scream variants produced in different emotional contexts. An analysis of the latter would require a larger sample of exemplars from each context. Overall, mean F0, duration, and roughness all significantly influenced participants’ ratings on at least one prompt; as explained earlier, mean HNR was excluded from acoustic models to avoid collinearity. Only F0 variation and loudness had no effect.

Many of the acoustic effects in our study were consistent with findings in prosodic speech and other nonlinguistic vocalizations. For example, higher scream F0 predicted higher fear ratings and, in other vocalizations, fear is often among the emotions associated with the highest F0 (Belin, Fillion-Bilodeau & Gosselin, 2008; Juslin & Laukka, 2003). It is notable, however, that this pattern holds in the perception of variation within screams, whereas in prior studies the correlation might have emerged because the fear stimuli, and only the fear stimuli, consisted of screams, which are characterized by higher pitch than other call types (Schwartz, Engelberg & Gouzoules, 2019). On the other hand, the lack of a role of F0 in the perception of any other emotion, or of any significant effect of F0 variation, contrasts with more general predictions (Scherer, 1986) and findings (Juslin & Laukka, 2003) that these parameters are broadly important for vocal emotion perception. F0 is most commonly implicated as a correlate of arousal (likely reflecting increased tension in the laryngeal musculature; Briefer, 2012), and pitch does contribute to listeners’ judgments of scream intensity (Schwartz & Gouzoules, 2019), but a single factor arousal framework does not seem to account for the present results: fear-associated contexts are not obviously linked to higher arousal than the other contexts in our study. It is perhaps relevant that fear perception in screams is not only semi-independent from the perception of other emotions, but also linked to such a salient vocal characteristic as F0. In keeping with the idea of emotion blends, listeners seemed to use F0 as a cue by which they sometimes perceived or did not perceive fear in addition to the concurrent perception of other emotions, a point highlighted by the fact that F0 was the strongest predictor not only of fear ratings but also of screams’ scores on the principal component mapping onto the perception of fear independently of other perceived emotions.

The effects of duration aligned reasonably well with the broader literatures on human and nonhuman emotional expression. Pain perception was associated with longer duration, whereas happiness and surprise perception were characterized by shorter duration. These results add further support to the positive relationship between duration and negatively-valenced states, which is one of the more consistently reported correlations across species and call types (Briefer et al., 2015, 2019; Friel et al., 2019; Raine et al., 2018; Scheiner et al., 2002; albeit with exceptions, for example, predator alarm calls are negative but characterized by short duration; Caro, 2005).

Roughness has garnered particular interest of late (Belin & Zatorre, 2015) because of the contention by Arnal et al. (2015) that, among human vocalizations, this feature is unique to screams, affording them a special acoustic niche for communicating alarm. Partially consistent with this hypothesis, we found that roughness positively predicted the perception of negative states, including anger, frustration, and pain, and negatively predicted the perception of happiness, although it did not significantly affect the perception of fear. These results suggest that, beyond its putative role in distinguishing screams acoustically from other call types, variation in roughness correlates with listeners’ perceptions of emotion among screams. Given that roughness variation in infant cries correlates with the vocalizer’s level of pain (Koutseff et al., 2018), it seems likely that roughness is an important cue not only for distinguishing call types associated with alarm, but also for interpreting relevant emotional variation within those call types. That said, in our study, roughness and mean HNR were highly correlated and accounted for similar variance in ratings, so it is difficult to discern the effects of one independent of the other. It is possible that the perception of noisiness, and not roughness *per se*, contributed to listeners’ perceptions of negative valence. Noisiness is sometimes, but not always, associated with increasing arousal (Briefer, 2012) or pain (Koutseff et al., 2018), but negatively correlated with pain or anger perception from other human nonlinguistic vocalizations (Lima, Castro & Scott, 2013; Raine et al., 2018). Nonlinear phenomena, such as chaotic noise produced by aperiodic vocal fold vibration, also result in less tonal sounds (Fitch, Neubauer & Herzel, 2002) and can lead to the perception of negative valence (Anikin, 2020), so the presence of these phenomena in our stimuli may have also played a role in our results.

Interestingly, our results show some parallels with the motivation-structural (MS) rules that Morton (1977) developed to account for variation in animal calls: namely, that hostile calls should converge upon a “harsh” (wideband, noisier) acoustic structure whereas calls functioning to appease conspecifics (e.g., those associated with friendly or fearful contexts) should sound more tone-like (as well as higher-pitched, consistent with the correlation between F0 and fear perception). Another study from our lab, examining acoustic variation in screams across macaque species, likewise provided nuanced support for MS rules (Gouzoules & Gouzoules, 2000).

Overall, our acoustic results are largely consistent with patterns seen across other mammalian vocalizations (Briefer, 2012; Briefer et al., 2019), human nonlinguistic vocalizations (Belin, Fillion-Bilodeau & Gosselin, 2008; Sauter et al., 2010), and speech prosody (Juslin & Laukka, 2003). These findings might suggest either that homologous mechanisms account for emotion-based variation in every case, leading to similar effects on perception, and/or that participants judged variation in screams based on their experience with other types of vocal expression, whether or not those judgments were accurate for screams.

### Accuracy and the case of happiness

Overall, participants were more likely to provide higher ratings on emotion prompts matching a scream’s original source context, circumspectly suggesting some degree of accuracy in listeners’ interpretations of screams. That said, this measure of accuracy did not necessarily entail precision, that is, ruling out all “incorrect” emotions (see the confusion of happiness-associated screams for fear, discussed in detail below), a determination made complicated in any event by the existence of emotion blends.

For the most part, our stimulus set was not sufficient to enable more detailed examination of accuracy by each source context. However, we provisionally investigated the interpretation of happiness screams as a special case, given that these were relatively well-represented among our stimuli and are potentially unique to humans. Strikingly, participants overall tended not to perceive happiness from our stimuli. Listeners instead provided high ratings of fear to happiness-associated screams, a finding that replicates earlier reports that both listeners (Anikin & Persson, 2017) and acoustic classifiers (Patel et al., 2011) often confuse intense joy for fear. In the section below, we offer a broader functional (and speculative) account for this confusion, but here we suggest a possible proximate factor: namely, general response biases towards fear and against happiness might suggest that listeners sometimes fell back on familiar stereotypes regarding the contexts in which screams occur. If this is the case, studies investigating scream perception across cultures or participant groups with relevant backgrounds (e.g., parents versus non-parents), whose stereotypes might vary or have changed through personal experience, could reveal somewhat robust percepts of happiness from screams. It is additionally likely that other contextual information (e.g., the social context; Wood, 2019) would modulate listeners’ interpretations of screams, and perhaps enable a confident assessment of a scream as joyful even if, absent contextual details, listeners might interpret it as fearful.

More broadly, it is possible that the imbalances in our stimulus set—namely, the relatively greater representation of happiness screams and of female versus male vocalizers—introduced subtle response biases that we did not detect. Although we found no evidence of any obvious such effects (e.g., differences in overall usage of emotion ratings in response to female- versus male-vocalized screams), we plan future studies that approach this issue with a larger and more balanced stimulus set.

### Function, emotion and diversity in screams

One perspective that is sometimes overlooked in studies of emotional expression is that, even in calls associated with highly emotional contexts, the communication of accurate or precise emotional information might not be the call’s only function, or, perhaps, even its most important. Consider, for example, that, at least in some social species, screams are probably selected to transmit over long distances, capture the attention of conspecific allies, and convey details other than the caller’s emotional state (e.g., identity-related attributes; Engelberg, Schwartz & Gouzoules, 2019). The acoustic and perceptual properties of screams are likely to reflect each of these functions, in turn bearing implications for emotion-related variation.

Indeed, conveying honest information about emotion may not always be beneficial, and may even be detrimental, to the caller and/or listener (an issue reviewed, in the broader context of animal communication, in Searcy & Nowicki, 2005). Callers in happy contexts might benefit by producing screams with the same acoustic characteristics that render other screams attention getting. As for listeners, to offer one possible account, it seems pertinent that screams associated with happiness or excitement are so prominent in childhood play (Sherman, 1975). Play might represent a safe context in which children can, in effect, “practice” screaming while parents and other kin can become familiar with the acoustic features distinguishing a given child’s screams from those of others (Engelberg, Schwartz & Gouzoules, 2019). It is thus perhaps functionally advantageous that these should resemble fear screams closely, such that, for example, parents might come to recognize their daughter screaming in genuine fear even if they had not heard her scream when truly frightened frequently. If those parents then sometimes over-attribute fear to happy screams (as our study participants did), absent additional contextual cues, the fitness consequences of this error would likely be relatively minor. Finally, the possibility that, in certain cases, other contextual cues might help listeners disambiguate fear and happiness may have further reduced selective pressures favoring discrimination from the screams’ acoustics alone, although it is important to note that screams often function in long-distance communication where a straightforward assessment of context is limited.

More broadly, the existence of screams associated with happiness or excitement, which seem unique to humans and thus might comprise an evolutionarily recent development, provokes questions regarding the expanded communicative potential of nonlinguistic communication in humans. No nonhuman primates scream in such heterogenous emotional contexts as humans. Screams have indeed functionally diversified in nonhuman primates, but they appear to have done so considerably further still in our species. It is probably no coincidence that a similar evolutionary trajectory is well-described in another human call type, laughter. In nonhuman species, laughter or laugh-like calls occur in a few very specific contexts, namely, tickle and/or play (Davila-Ross, Owren & Zimmermann, 2009; Burgdorf et al., 2007; van Hooff, 1972). Humans, on the other hand, laugh in profoundly diverse and socially intricate situations (Gervais & Wilson, 2005; Provine, 2000).

In humans, then, there emerges a trend whereby the range of emotional and social contexts in which particular nonlinguistic calls occur has expanded (even if the size of the overall repertoire may have decreased; Deacon, 1998). Some language theorists refer to language’s derived functions, or its secondary uses apart from the direct functions for which it was selected (Oesch, 2016). We suggest that language and its cognitive underpinnings have effected inextricable changes on the nonlinguistic call system. These changes might include, but are not limited to, increased flexibility of vocal production (Pisanski et al., 2016); enhanced abilities of (and natural inclinations towards) meaning attribution (Scott-Phillips, 2015); and further emancipation from emotion-based calling (Oller et al., 2013), to say nothing of instances where natural calls and language co-occur (e.g., screamed or laugh-like speech; Menezes & Igarashi, 2006). Within preexisting call types such as screams, vocalizers might introduce new variation that conveys novel meanings in different contexts, and listeners might learn to derive those meanings and reproduce the variation in their own calls. That is, although nonlinguistic vocalizations differ functionally and evolutionarily from speech, they nonetheless may interact with language in deep-seated ways, and sometimes, a “nonlinguistic” call might function not unlike a conventionalized word. In other words, the diversification and enhancement of the information provided by nonlinguistic calls might represent a derived or secondary function of the language faculties (Oesch, 2016).

## Conclusion

Our findings indicate that listeners can perceive different emotional content from human screams. Many of these perceptions were evidently based on similar acoustic cues as those involved in other types of vocal emotional communication, but the effects of some parameters hint at potentially interesting aspects of variation in screams that require further investigation. Listeners tended to provide higher ratings on emotion prompts matching screams’ original source context, suggesting that acoustic variation among screams conveys some relevant information about variation in context. However, an examination of happiness screams revealed that they were not accurately perceived, and more commonly yielded higher ratings of fear. The constraints of variation within a call type and the functions of a call type other than emotional communication are likely significant to interpreting these results. Speculatively, both the contextual and acoustic diversification of screams and other nonlinguistic vocalizations might have emerged partly through a process of coevolution with language.

## Supplemental Information

10.7717/peerj.10990/supp-1 Supplemental Information 1 Raw data of participants’ responses. Participants’ ratings on every emotion prompt to every stimulus.

10.7717/peerj.10990/supp-2 Supplemental Information 2 Mean ratings and acoustic data for each stimulus.

## Additional Information and Declarations

### Data Availability

The following information was supplied regarding data availability: Raw data is available in the Supplemental Files.
